# Limitations in a rapid environmental scan of global health research expertise point to the need for more open data

**DOI:** 10.1186/s12961-020-00635-4

**Published:** 2020-11-03

**Authors:** Ranjana Nagi, Susan Rogers Van Katwyk, Steven J. Hoffman

**Affiliations:** 1 Global Strategy Lab, Dahdaleh Institute for Global Health Research, Faculty of Health, and Osgoode Hall Law School, York University, 4700 Keele Street, 2120 Dahdaleh Building, Toronto, Ontario M3J 1P3 Canada; 2 Department of Health Research Methods, Evidence and Impact and McMaster Health Forum, Faculty of Health Sciences, McMaster University, Hamilton, Ontario Canada

We thank Gyorkos [1] for commenting on our rapid environmental scan of global health research expertise in Canada [2]. Gyorkos argues that our scan was not comprehensive because we used too few data sources to measure Canadian global health research inputs, activities and outputs. Limitations in data, which were noted in our published study [2], highlight the current challenge of conducting rapid research within short policy windows using publicly available data sources in Canada and point towards opportunities for improving data infrastructure across countries.

First, in line with our rapid approach, we made choices that resulted in what we believe to be the most rigorous environmental scan possible within the available resources and a set 2-month policy window. This meant that we were limited to drawing on publicly available administrative datasets and were prevented from collecting new data. For global health research inputs, we focused our analysis on data from Canada’s largest funder of global health research – the Canadian Institutes of Health Research – as other Canadian funding agencies do not make their global health research funding data readily available. Gyorkos additionally flags our omission of funding data from the Bill and Melinda Gates Foundation, the Global Fund to Fight AIDS, Tuberculosis and Malaria, WHO, and UNAIDS, yet none of these entities make their funding data available or searchable by location. While the Wellcome Trust makes this data available, from 2005 to 2018 they awarded only six grants to researchers at Canadian universities totalling £544,966, representing a tiny fraction of Canada’s global health research funding landscape [3]. This challenge points to a need for all funding organisations to not only make their data more open but to ensure that data is collected, catalogued and disseminated in ways that enables external analysis, as the Canadian Institutes of Health Research and the Wellcome Trust have done. More open data would support rapid environmental scans like ours and inform the optimal allocation of limited resources by a range of stakeholders.

Second, our short 2-month policy window also necessitated choices about which key indicators of Canadian global health research activities would be relied upon. We focused on (1) academic research training programmes, (2) Canada Research Chairs and (3) global health research centres, including WHO Collaborating Centres. As noted in our study, these indicators of activity are not exhaustive; indeed, the environmental scan would benefit, for example, from including public events such as global health conferences and workshops. Future environmental scans can also make use of any existing lists of global health experts such as the Canadian Women in Global Health list [4] that Gyorkos cites but which had not yet been published at the time of our study.

Third, for global health research outputs, conducting a systematic bibliometric analysis of a whole country’s publication record would have taken us beyond the 2-month timeframe we had available to inform the policy decision for which we undertook this rapid environmental scan. Our use of the MEDLINE/PubMed ‘global health’ MeSH terms allowed us to include some research outputs in our rapid scan, in addition to research inputs and activities. Like Gyorkos, we acknowledge in our study that a more optimised search for publications authored by global health researchers at Canadian institutions would have been better but such a search was beyond what we could do within our set timeline. This points to the need to develop and validate optimised search strategies and comprehensive catalogues for global health research, as has been done for other fields [5]. However, such an effort will be complicated by the lack of an operational definition of ‘global health’ and disagreement on what the field encompasses [6, 7].

Like most countries, Canada would benefit from a more comprehensive analysis of its global health research expertise. Our study was purposefully and strategically designed to be rapid and to assess what we could describe within a 2-month timeline. We stand by the choices we made to accommodate this timeline. Knowing that future policy windows are likely to be similarly short, we suggest that critiques would be more productively directed towards those research funders and global health entities who have not yet made their data publicly available and thereby prevent more comprehensive scans in short timeframes. We certainly support open and transparent data-sharing by all entities whenever legally and ethically possible.

## Data Availability

Not applicable.
